# Risk factors for metachronous esophageal squamous cell carcinoma after endoscopic or surgical resection of esophageal carcinoma: a systematic review and meta-analysis

**DOI:** 10.3389/fonc.2023.1241572

**Published:** 2023-09-14

**Authors:** Jie Du, Zhixian Bao, Tianhu Liang, Hongmei Zhao, Junxian Zhao, Ruipu Xu, Xiaohui Wang

**Affiliations:** ^1^ Department of Social Medicine and Health Management, School of Public Health, Lanzhou University, Lanzhou, China; ^2^ The First Clinical Medical College, Lanzhou University, Lanzhou, China; ^3^ Department of Gastroenterology, The First Hospital of Lanzhou University, Lanzhou, China; ^4^ Key Laboratory for Gastrointestinal Diseases of Gansu Province, The First Hospital of Lanzhou University, Lanzhou, China; ^5^ Talent Service Center, Gansu Provincial Health Commission, Lanzhou, China

**Keywords:** metachronous esophageal squamous cell carcinoma, esophageal carcinoma, risk factors, second primary carcinoma, meta-analysis

## Abstract

**Background:**

early-stage esophageal carcinoma (EC) patients lack typical clinical signs and symptoms and are often diagnosed and treated at a late stage, leading to a poor prognosis and a high incidence of metachronous esophageal squamous cell carcinoma (MESCC) and second primary carcinoma (SPC). The aims of the review were to identify and quantify risk factors for MESCC and analysis location of SPC in postoperative patients with EC; to predict incidence of MESCC over follow-up time.

**Methods:**

an electronic search of studies reporting potential risk factors, the incidence of MESCC, and the location of SPC were performed on PubMed, Web of Science, Cochrane Library, Embase, and Scopus from inception to 10 November 2022. The Newcastle-Ottawa scale was used to assess the study quality, and the qualitative strength of evidence rating of all items was provided. The meta-regression model was used to predict the incidence of MESCC over follow-up time, the location distribution of SPC was presented using clustered column chart, while the publication bias was assessed using funnel plots and Egger’s test.

**Results:**

smoking, age, history of multiple other cancer, and Lugol-voiding lesions (LVLs) were determined to be the risk factors of MESCC. LVLs were qualitatively determined as “definite” and the history of multiple other cancer as “likely.” The overall pooled MESCC incidence was 20.3% (95% CI: 13.8% to 26.8%), with an increase of 0.20% for each additional year of follow-up. The head and neck were the most common locations for SPC, followed by the esophagus.

**Conclusion:**

timely investigating the age of patients, previous history of cancer and monitoring the number of LVLs in the first 5 years after operation are of great significance to identify high-risk populations of MESCC for timely medical care. Education and behavior correction about smoking are advocated. Tumor markers should be regularly detected in the head and neck, esophagus, and stomach. Endoscopic resection was associated with a higher incidence of MESCC, which provided a reference for doctors to choose the removal method.

**Systematic review registration:**

https://www.crd.york.ac.uk/PROSPERO/, identifier CRD42022377030.

## 1 Introduction

Esophageal carcinoma (EC) is a significant health issue ranked the eighth most prevalent globally. The 5-year survival rate range from 15-25%; thus, it is the sixth most common cause of cancer-related deaths in the world (1). Esophageal squamous cell carcinoma (ESCC) is the most prominent globally among esophageal cancers. The “Asian esophageal carcinoma belt” consisting of Iran, Kazakhstan, and northern and central China has one of the highest incidences of ESCC, estimated to exceed 100 cases per 100,000 persons annually. Southeastern Africa and the United States are other regions with high incidence (1, 2). Currently, endoscopic resection (ER) and esophagectomy are the first and second treatment options for EC in Western countries and Asia, primarily Japan and China. The ER is considered a first-line EC treatment because it is minimally invasive (3). However, patients with early-stage EC lack typical clinical signs and symptoms and are often diagnosed late, making the outcome of EC treatment poor. Moreover, EC patients are likely to develop MESCC after treatment, which makes the survival and prognosis of patients even worse. Metachronous ESCC (MESCC) is an independent primary malignancy that arises in the preserved esophagus after the first endoscopic or surgical treatment of EC. Some studies have identified the Lugol-voiding lesions (LVLs) as a risk factor for MESCC (4, 5). In contrast, others have associated age with MESCC (6–8), but there are no relevant reviews or meta-analyses on the risk factors of MESCC. Therefore, it is imperative to identify and systematically summarize the risk factors for MESCC for optimal prevention, surveillance, prompt diagnosis, treatment, and post-treatment follow-up.

Studies have shown varying incidences of MESCC after treatment. A study by Uno et al. reported an incidence of 14.9% in 40.5 months after endoscopic submucosal dissection (9), while Kim et al. reported about 8.3% in 6 years after endoscopic submucosal dissection (10). Furthermore, the incidence of MESCC has been reported to increase over time, from 11.4%, 20.6%, and 39.3% in 2, 5, and 10 years after endoscopic submucosal dissection, respectively (7). However, no unified conclusion exists on the incidence of MESCC after ER for EC. Thus, evaluating the cumulated MESCC incidence and predicting the 5-year and 10-year incidence rates is necessary. In addition, it has been reported that an unrelated second primary carcinoma (SPC) could occur in treated EC patients. The SPC is a primary cancer occurring at any site after endoscopic or surgical treatment of EC and pathologically proven not to be the recurrence or metastasis of the first carcinoma. According to studies (11–13), SPC after endoscopic or surgical treatment for EC may occur in the stomach, esophagus, liver, lung, and lymph nodes. Nevertheless, there are limited knowledge about their distribution among studies. Therefore, it is necessary to comprehensively describe the location of SPC after endoscopic or surgical treatment for EC.

This study explored the risk factors for the development of MESCC, evaluated the incidence of MESCC, and ranked the locations of the SPC. The outcome of this analysis will guide clinical practices and effectively identify high-risk populations, optimizing the outcome of patient prognosis.

## 2 Methods

### 2.1 Protocol

The protocol was registered according to the Preferred Reporting Items for Systemic Reviews and Meta-Analysis (PRISMA) guidelines (14, 15) in the International Prospective Register of Systematic Reviews (PROSPERO) with registration number CRD42022377030.

### 2.2 Search strategy

With the assistance of an evidence-based medicine specialist, the following search terms of interest were considered to construct a strategy: (1) the esophagus, (2) the method of removing carcinoma, (3) carcinoma occurrence after removal, and (4) risk factors. The online databases of PubMed, Web of Science, Cochrane Library, Embase, and Scopus were searched from inception to 10 November 2022. The search terms were performed separately in all databases and combined with free words. Furthermore, the reference lists of all included articles were screened manually to capture studies omitted in the initial search. Only literature published in English without regional restrictions was included. The search strategies on all databases are presented in the **Supplementary Materials** (Search Strategies).

### 2.3 Eligibility criteria

MESCC was defined as a non-recurrent squamous cell carcinoma that appeared in the esophagus after the first endoscopic or surgical treatment of EC. SPC was defined as primary carcinoma occurring at any site after endoscopic or surgical treatment of EC, which was pathologically proven not to be the recurrence or metastasis of the first carcinoma. Inclusion criteria were: 1) patients with curative ER (endoscopic submucosal dissection and endoscopic mucosal resection) or surgery (esophagectomy) or the above regiments combined with chemoradiotherapy, excluding chemoradiotherapy alone and untreated, 2) intervention: existed a risk factor associated with the occurrence of MESCC, 3) comparison: without the risk factor above, 4) reported at least 1 potential risk factor or incidence of MESCC or distribution of SPC and met the definition, 5) randomized controlled trials, cohort or case-control studies. Exclusion criteria were: 1) studies with duplicate data (choose the study with the most comprehensive data), 2) case reports, reviews, comments, and meeting records, 3) animal studies and 4) publications on other diseases.

### 2.4 Study selection

The screening process was conducted by automatically excluding duplicated studies using EndNote X9 software and manually by a reviewer. Afterward, the titles, abstracts, and full text were reviewed independently by 2 reviewers according to the inclusion and exclusion criteria, and discrepancies were resolved by consensus or by a third independent researcher. All relative studies that addressed complications other than metachronous carcinoma and second carcinomas after EC removal were excluded during the title and abstract review phase. Case reports, case series, and meeting abstracts were excluded. Prospective studies can identify potentially modifiable risk factors because the sequence of items and the development of MESCCs is clear. Conversely, items identified in the retrospective study can only be considered associated modifiable factors because of no temporal relationship.

### 2.5 Quality assessment

The semi-quantitative principle of star system of the Newcastle-Ottawa Scale (NOS) was used to assess the quality of observational studies. The risk of bias and methodological quality of the included studies was assessed independently by 2 blinded reviewers, and discrepancies were resolved through discussions with a third reviewer. The NOS tool consists of 8 items in 3 domains (selection, comparability, and outcome) used to assess the quality of cohort studies. For each item, a series of response options are provided with a star system such that higher stars indicate higher quality. Based on the full-text review of each study, the researcher must select the most appropriate response option. If responses indicate a low risk of bias, 1 star is assigned, except for the comparability domain, which is assigned 2 stars. Overall, the score ranges from 0 – 9 stars, with 1-4 stars, 5-6 stars, and 7-9 stars indicating low, moderate, and high quality, respectively. Studies were not excluded based on their quality assessment score. Studies with low-quality assessment scores were not excluded. However, their score was used to determine the strength of their evidence qualitatively.

### 2.6 Data extraction

Data were collected in a standard pre-designed form and verified independently by reviewers. The data include 1) The baseline characteristics comprising title, first author, publication year, region, study design, study period, sample size, inclusion and exclusion criteria, 2) risk factors: removal method of EC, the definition of MESCC, follow-up period, potential risk factor, the method of factor measurement, measurement values reported, statistical test used, significance level; 3) incidence of MESCC, the definition of SPC, location of SPC; 4) methodological information: all relevant details on selection, comparability, and outcome or exposure. Studies with the same subjects or a part of them were excluded to avoid duplication. Some risk factors that fall into more than 2 categories, such as alcohol consumption, were regrouped into 2, rarely drinking and heavy drinking. However, in the original text, there were four groups: seldom, light, moderate, and heavy drinking. Raw data and hazard ratio were recorded for each risk factor when available. Adjusted effect sizes were preferred, followed by raw data if the above data were present simultaneously.

### 2.7 Data synthesis and analysis

The risk ratio (RR) at 95% confidence interval (CI) for factors of dichotomous variables was calculated using the Comprehensive Meta-Analysis software (version 3.3.070) and the statistical significance (p-value) at <0.05. The pooled effect sizes were computed based on the heterogeneity among studies, with the fixed effects model when it was low and the random effects model when it was high. Heterogeneity was evaluated using I^2^ statistics and Cochran’s Q test with a value of >50% and/or p<0.05, indicating significant heterogeneity. Subgroup analysis was performed to explore potential sources of heterogeneity according to a) removal method, b) type of cohort study (prospective or retrospective), c) follow-up schedule (annual or biannual or every 6 – 12 months), d) median/mean follow-up time (< or >60 months), and e) data type. Sensitivity analysis was performed by applying the other analysis model or (and) excluding 1study at a time. Risk factors reported by 3 or more studies were quantitatively analyzed to estimate a combined RR. Likewise, the strength of evidence of all items was qualitatively rated to label each risk factor as “definite,” “likely,” “unclear,” or “not a risk factor” based on the number and percentage of studies evaluating the factors and showing a positive association (**Table 1**). The pooled incidence of MESCC was calculated with a random-effects model. Using R software (version 4.1.3), the Meta-regression model predicted MESCC occurrence over follow-up time at yearly intervals. The location distribution of SPC was presented using a clustered column chart. The funnel plots and Egger’s test were used to qualitatively and quantitatively assess publication bias. Funnel plots were applied for the outcomes of more than 5 enrolled studies.

**Table 1:** Defining the strength of a risk factor.

Strength of evidence	Applicable condition
Definite	All studies of moderate and high quality positive (at least 3 studies); Majority (more than 50%) studies of moderate and high quality positive (at least 5 studies).
Likely	All studies of moderate and high quality positive (2 studies); Majority (more than 50%) studies of moderate and high quality positive (2-4 studies).
Unclear	All studies of moderate and high quality positive (1 study); Studies of moderate and high quality show mixed or conflicting results; A majority (more than 50%) of studies negative but at least 1 study of moderate and high quality positive.
Not a risk factor	No studies of moderate and high quality positive

## 3 Results

### 3.1 Study selection

**Figure 1:**
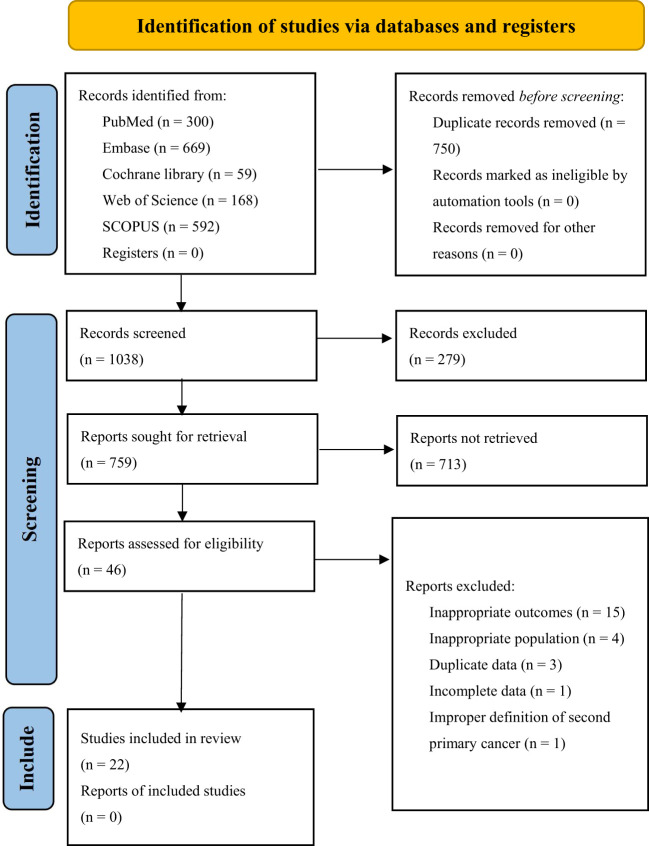
Flow diagram of the selection process for the studies.

The initial 5 database searches yielded a total of 1788 articles. After EndNote X9 software automatically removed duplicates, 1038 articles remained. Upon screening the titles and abstracts of the 1038 articles, only 92 studies were included for full-text review. Following the full-text review, 22 studies were included in this study, among which 13 were analyzed for risk factors, 21 for incidence of MESCC, and 9 studies for the location distribution of SPC. A total of 13 factors, 11 of which were quantitatively analyzed. The **Supplementary Materials** (**Figure 1**) shows the flowchart illustrating the retrieved articles’ selection process.

### 3.2 Study characteristics

The characteristics of included studies and quality evaluation are displayed in **Supplementary Materials (Table 2)** and **Table 2**. All the studies utilized a cohort study design, of which 17 were retrospective and 5 prospective. The studies were performed in Asia, with the majority from Japan, 2 from mainland China, 2 from Taiwan, China and 1 from Korea. The ESCC was the most common type of pathological EC reported in most (18/22) of the studies consisting of 5151 patients, while only 4 studies with 1191 patients reported adenocarcinoma, precancerous lesion, intra-mucosal, or unclassified carcinoma. Among the patients in the included studies, 3651 (16 studies), 2323 (5 studies), and 368 (1 study) patients underwent ER of EC, surgery, and ER or surgery, respectively. The follow-up time was reported in different forms, of which 14 studies were reported in median, 4 in mean, and the remaining 4 in other forms or unknown.

**Table 2:** Quality Evaluation of Included Studies.

Study	Selection				Comparability	Outcome			Total scores	Study quality
	Representative of the exposed cohort	Selection of the non-exposed cohort	Ascertainment of exposure	Demonstration that outcome of interest was not present at start of study	Comparability of cohorts on the basis of the design or analysis	Assessment of outcome	Follow-up long enough for outcomes to occur	Adequacy of follow up of cohorts		
Satoshi Abiko 2018 (16)	1	1	1	1	1	1	1	1	8	High
Yuka Azuma 2022 (17)	1	1	1	1	2	0	1	1	8	High
Kenichi Kagemoto 2016 (18)	1	1	1	1	0	0	1	1	6	Moderate
Nobuhiko Ogasawara 2021 (7)	1	1	1	1	1	1	1	1	8	High
Ga Hee Kim 2020 (10)	1	1	1	1	2	0	1	1	8	High
Yuji Urabe 2018 (19)	1	1	1	1	2	0	1	1	8	High
Wen-Lun Wang 2016 (8)	1	1	1	1	0	1	1	1	7	High
Ming‐Hung Hsu 2021 (20)	1	1	1	1	0	1	1	1	7	High
Ayaka Tajiri 2022 (21)	1	1	1	1	1	1	1	1	8	High
Y. Otowa 2015 (22)	1	1	1	1	1	0	1	1	7	High
Dongxian Jiang 2016 (23)	1	1	1	1	0	1	1	1	7	High
A.Kokawa 2001 (24)	1	1	1	1	2	0	1	1	8	High
Ichiro Oda 2020 (25)	1	1	1	1	0	0	1	1	6	Moderate
Toshiyuki Yoshio 2022 (26)	1	1	1	1	2	1	1	1	9	High
Wen-Si Hu 2015 (12)	1	1	1	1	1	0	1	1	7	High
Toshiki Matsubara 2003 (27)	1	1	1	1	2	0	1	1	8	High
Kengo Onochi 2018 (28)	1	1	1	1	1	1	1	1	8	High
K. Uno 2017 (9)	1	1	1	1	2	0	1	1	8	High
Yusuke Sato 2005 (29)	1	1	1	1	0	0	1	1	6	Moderate
Akira Maekawa 2019 (30)	1	1	1	1	0	0	1	1	6	Moderate
Chikatoshi Katada 2016 (31)	1	1	1	1	1	0	1	1	7	High

### 3.3 Risk factors

#### 3.1.1 Demographic, individual factors

Smoking and gender were quantitatively analyzed in 10 studies with low heterogeneity (I^2^ = 40.20% and 38.99%, respectively). The risk of MESCC was positively correlated with the amount of smoking (RR1.504, 95%CI 1.175 to 1.924, P=0.001) and had no statistically significant association with gender (RR0.871, 95%CI 0.688 to 1.102, P=0.251). The results of fixed effects model and random effects model were statistically consistent. The results of the subgroup analysis based on pre-determined subgroup variables were presented in **Table 3**. The results indicated that the follow-up time was a source of heterogeneity for smoking as a potential risk factor. Upon dividing the follow-up time into 3 groups, the heterogeneity of all the groups were 0. However, only the unknown follow-up time group with a negative result was statistically insignificant. Neither the subgroup analysis nor the sensitivity analysis identified sources of heterogeneity.

**Table 3:** Subgroup analysis of potential risk factors with high heterogeneity.

Potential risk factors	Subgroup Category	Subgroup	Number of studies	Heterogeneity (I^2^, %)	RR (95%CI)
Smoking	RM	ER	6	6.64	1.33 (1.00, 1.76)
		CRT after ER	3	0	3.21 (1.79, 5.75)
		Surgery/ER	1	0	0.83 (0.32, 2.12)
	TCS	prospective	1	0	3.07 (1.17, 8.06)
		retrospective	9	37.46	1.43 (1.11, 1.85)
	FT	<60 months	3	0	3.14 (1.76, 5.58)
		>60 months	5	0	1.43 (1.06, 1.93)
		–	2	0	0.77 (0.41, 1.45)
	FS	Annual	1	0	3.07 (1.17, 8.06)
		Biannual	3	10.44	1.07 (0.65, 1.76)
		Every6-12 months	5	0	1.42 (1.04, 1.94)
		–	1	0	4.44 (1.71, 11.58)
	DT	Raw	7	48.52	1.69 (1.25, 2.29)
		HR	3	0	1.19 (0.78, 1.82)
Gender	RM	ER	6	50.98	0.83 (0.64, 1.07)
		CRT after ER	3	45.12	1.14 (0.58, 2.22)
		Surgery/ER	1	0	1.18 (0.28, 4.96)
	TCS	prospective	1	0	2.54 (0.67, 9.56)
		retrospective	9	34.34	0.84 (0.66, 1.07)
	FT	<60 months	3	65.83	0.58 (0.35, 0.96)
		>60 months	5	22.27	0.99 (0.75, 1.30)
		–	2	0	0.83 (0.31, 2.22)
	FS	Annual	1	0	2.54 (0.67, 9.56)
		Biannual	3	2.55	1.14 (0.60, 2.19)
		Every6-12 months	5	49.3	0.82 (0.63, 1.06)
		–	1	0	0.29 (0.05, 1.74)
	DT	Raw	6	34.49	0.84 (0.65, 1.08)
		HR	4	53.82	1.09 (0.60, 1.99)
Alcohol consumption	RM	ER	3	44.37	1.44 (0.83, 2.51)
		CRT after ER	2	0	12.36 (2.53, 60.48)
		Surgery/ER	1	0	0.54 (0.21, 1.36)
	FT	<60 months	2	85.26	1.40 (0.72, 2.73)
		>60 months	3	0	2.76 (1.19, 6.42)
		–	1	0	0.54 (0.21, 1.36)
	FS	Biannual	1	0	7.52 (0.48, 117.78)
		Every6-12 months	4	55.67	1.11 (0.69, 1.79)
		–	1	0	15.84 (2.27, 110.73)

RR, risk ratio; RM, removal method; ER, endoscopic resection; CRT, chemoradiotherapy; TCS, type of cohort study; FT, follow-up time; -, missing data; FS, follow-up schedule; DT, data type; HR, Hazard Ratio.

There was no heterogeneity among the 6 articles analyzed for age as a potential risk factor. However, the study of Wen-Lun Wang (8) set 50 years old as the cut-off value, while others reported 65 years. Nevertheless, the result was still statistically significant after excluding this study, and the risk of MESCC increased with younger age (RR 0.690, 95% CI 0.564 to 0.844, p=0.000).

The 6 studies about alcohol consumption were enrolled with high heterogeneity (I^2^ = 66.817%). There was no significant association between alcohol consumption and MESCC in the fixed (RR 1.356, 95% CI 0.859 to 2.141, p=0.191) and random (RR 2.091, 95% CI 0.843 to 5.190, p=0.112) effect models. Subgroup analysis did not find out a source of heterogeneity. In sensitivity analysis, after excluding of the study of Yuka Azuma (17), the results became to be statistically significant (RR3.014, 95%CI 1.105 to 8.223, P=0.031). However, the heterogeneity remained almost unchanged.

The 4 studies that described the history of multiple other cancers as a risk factor had a low heterogeneity (I^2 ^= 2.925%). The study participants with a history of multiple other cancer were more likely to develop MESCC (RR 2.089, 95% CI 1.494 to 2.921, p=0.000). Three studies reported smoking after ER as a potential risk factor for MESCC. However, it was not statistically significant (RR 1.165, 95% CI 0.744 to 1.823, p=0.505). Also, the 3 studies showed no heterogeneity.

**Figure 2:**
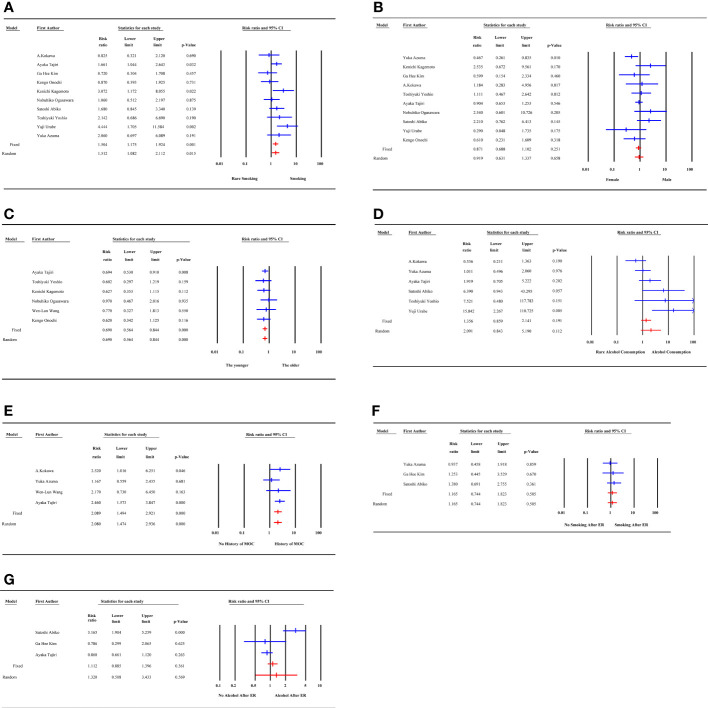
Forest plot of demographic, individual factors. **(A)** smoking; **(B)** gender; **(C)** age; **(D)** alcohol consumption; **(E)** history of multiple other cancer; **(F)** smoking after ER; **(G)** alcohol consumption after ER; MOC, multiple other cancer; ER, endoscopic resection.

The 3 studies that described alcohol consumption after ER as a risk factor had a high heterogeneity (I^2 ^= 90.207%). However, there was no statistical significance and heterogeneity when the study by Satoshi Abiko (16) was excluded in the sensitivity analysis. The results showed no significant association between alcohol consumption after ER and MESCC (RR 1.320, 95% CI 0.508 to 3.433, p=0.569), as shown in **Figure 2**.

Qualitatively, the studies that reported risk factors were of moderate or high quality with smoking, gender, age, alcohol consumption, history of multiple other cancer, smoking after ER, and alcohol consumption after ER showing 3, 1, 1, 1, 2, 0, and 1 positive results, respectively. Most of the strength of evidence for the risk factors was labeled as unclear, 1 as likely, and 1 as not a risk factor qualitatively in **Tables 4** and **5**.

**Table 4:** Qualitative and quantitative results of risk factors and incidence analysis.

Potential factors	Number of studies	Quantitative results		Qualitative results	MEC/total (%)
		I^2^ (%)	RR (95%CI)		
Demographic, individual factors					
Smoking	10	40.20	1.504(1.175,1.924)*	Unclear	436/2516
Gender	10	38.99	0.871(0.688,1.102)	Unclear	456/2007
Age	6	0	0.690(0.564,0.844)*	Unclear	319/1109
Alcohol consumption	6	66.82	2.091(0.843,5.190)	Unclear	299/1221
History of multiple other cancer	4	2.93	2.089(1.494,2.921)*	Likely	200/876
Smoking after ER	3	0	1.165(0.744,1.823)	Not a risk factor	98/513
Alcohol consumption after ER	3	90.21	1.320(0.508,3.433)	Unclear	195/704
Endoscopic and histological factors					
LVLs	9	0	3.667(2.587,5.197)*	Definite	414/1594
Tumor location	4	0	1.095(0.835,1.435)	Not a risk factor	235/736
Macroscopic type	4	40.95	0.976(0.636,1.498)	Not a risk factor	288/813
Tumor depth	3	0	0.736(0.395,1.369)	Not a risk factor	92/522
Tumor size	2	–	–	Not a risk factor	98/255
Lymphovascular invasion	2	–	–	Not a risk factor	62/420

RR, risk ratio; MEC, metachronous esophageal carcinoma; ER, endoscopic resection; LVLs, Lugol-void ing lesions; -, missing data; * statistical significance.

**Table 5:** Quantitative results and data types of potential risk factors.

Potential factors	Study	Study quality	Positive results	Data type
Demographic, individual factors				
Smoking	A.Kokawa (24)	High	No	Raw
	Ayaka Tajiri (21)	High	Yes	Raw
	Ga Hee Kim (10)	High	No	Raw
	Kengo Onochi (28)	High	No	HR
	Kenichi Kagemoto (18)	Moderate	Yes	Raw
	Nobuhiko Ogasawara (6)	High	No	HR
	Satoshi Abiko (16)	High	No	HR
	Toshiyuki Yoshio (26)	High	No	Raw
	Yuji Urabe (19)	High	Yes	Raw
	Yuka Azuma (17)	High	No	Raw
Gender	Yuka Azuma (17)	High	Yes	Raw
	Kenichi Kagemoto (18)	Moderate	No	Raw
	Ga Hee Kim (10)	High	No	Raw
	A.Kokawa (24)	High	No	Raw
	Toshiyuki Yoshio (26)	High	No	Raw
	Ayaka Tajiri (21)	High	No	Raw
	Nobuhiko Ogasawara (7)	High	No	HR
	Satoshi Abiko (16)	High	No	HR
	Yuji Urabe (19)	High	No	HR
	Kengo Onochi (28)	High	No	HR
Age	Ayaka Tajiri (21)	High	Yes	Raw
	Toshiyuki Yoshio (26)	High	No	Raw
	Kenichi Kagemoto (18)	Moderate	No	Raw
	Nobuhiko Ogasawara (7)	High	No	HR
	Wen-Lun Wang (8)	High	No	HR
	Kengo Onochi (28)	High	No	HR
Alcohol consumption	A.Kokawa (24)	High	No	Raw
	Yuka Azuma (17)	High	No	Raw
	Ayaka Tajiri (21)	High	No	Raw
	Satoshi Abiko (16)	High	No	Raw
	Toshiyuki Yoshio (26)	High	No	Raw
	Yuji Urabe (19)	High	No	Raw
History of multiple other cancer	A.Kokawa (24)	High	Yes	Raw
	Yuka Azuma (17)	High	No	Raw
	Wen-Lun Wang (8)	High	No	HR
	Ayaka Tajiri (21)	High	Yes	HR
Smoking after ER	Yuka Azuma (17)	High	No	Raw
	Ga Hee Kim (10)	High	No	Raw
	Satoshi Abiko (16)	High	No	HR
Alcohol consumption after ER	Satoshi Abiko (16)	High	Yes	Raw
	Ga Hee Kim (10)	High	No	Raw
	Ayaka Tajiri (21)	High	No	Raw
Endoscopic and histological factors				
LVLs	Yuka Azuma (17)	High	Yes	Raw
	Kenichi Kagemoto (18)	Moderate	No	Raw
	Ga Hee Kim (10)	High	No	Raw
	Yuji Urabe (19)	High	Yes	Raw
	Ayaka Tajiri (21)	High	Yes	Raw
	Toshiyuki Yoshio (26)	High	No	Raw
	Nobuhiko Ogasawara (7)	High	Yes	HR
	Wen-Lun Wang (8)	High	Yes	HR
	Kengo Onochi (28)	High	Yes	HR
Tumor location	Yuka Azuma (17)	High	No	Raw
	Ga Hee Kim (10)	High	No	Raw
	Ayaka Tajiri (21)	High	No	Raw
	Nobuhiko Ogasawara (7)	High	No	HR
Macroscopic type	Yuka Azuma (17)	High	No	Raw
	Nobuhiko Ogasawara (7)	High	No	HR
	Ayaka Tajiri (21)	High	No	Raw
	Chikatoshi Katada (31)	High	No	HR
Tumor depth	Yuka Azuma (17)	High	No	Raw
	Ga Hee Kim (10)	High	No	Raw
	Ming‐Hung Hsu (20)	High	No	HR
Tumor size	Nobuhiko Ogasawara (7)	High	No	HR
	Ming‐Hung Hsu (20)	High	No	HR
Lymphovascular invasion	Ga Hee Kim (10)	High	No	HR
	Ming‐Hung Hsu (20)	High	No	HR

HR, Hazard Ratio; ER, endoscopic resection; LVLs, Lugol-voiding lesions.

#### 3.3.2 Endoscopic and histological factors

**Figure 3:**
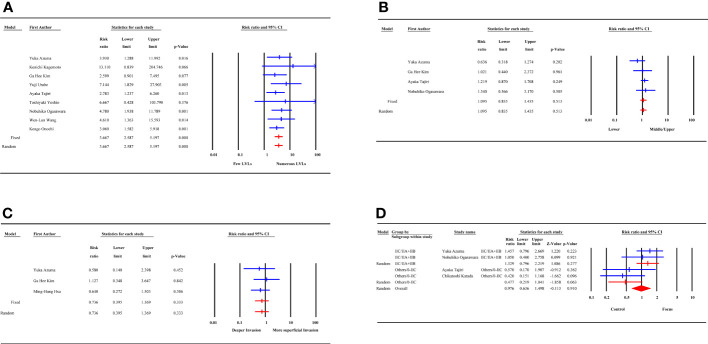
Forest plot of endoscopic and histological factors. **(A)** LVLs; **(B)** tumor location; **(C)** tumor depth; **(D)** macroscopic type; LVLs, Lugol-voiding lesions.

The 9 LVLs, 4 tumor location, and 3 tumor depth studies had no heterogeneity. The risk of MESCC was associated with increasing LVLs (RR 3.667, 95% CI 2.587 to 5.197, p=0.000), irrespective of tumor location (RR 1.095, 95% CI 0.835 to 1.435, p=0.513) and tumor depth (RR 0.736, 95% CI 0.395 to 1.369, p=0.333). Based on different cut-off standards, the subgroup analysis of the 4 studies on the macroscopic type of tumor showed a decrease in the source of heterogeneity from 49.5% to 0%. However, the results for both subgroups were not statistically significant, which was consistent with the overall result, as shown in **Figure 3**.

Qualitatively, the studies that reported risk factors were of moderate or high quality. Except for 6 studies on LVLs, none of studies on the tumor location, tumor depth and macroscopic type showed positive results. The above 3 factors were labeled as not a risk factor except for LVLs, whose strength of evidence was qualitatively definite. Two studies identified tumor sizes with a cut-off value of 35mm or 30 mm as a factor; however, there was no significant relationship between tumor size and MESCC. Two studies reported lymphovascular invasion as a factor and were not statistically significant. All qualitative and quantitative results are summarized in **Tables 4** and **5**.

### 3.4 Incidence of MESCC after ER and surgery

**Figure 4:**
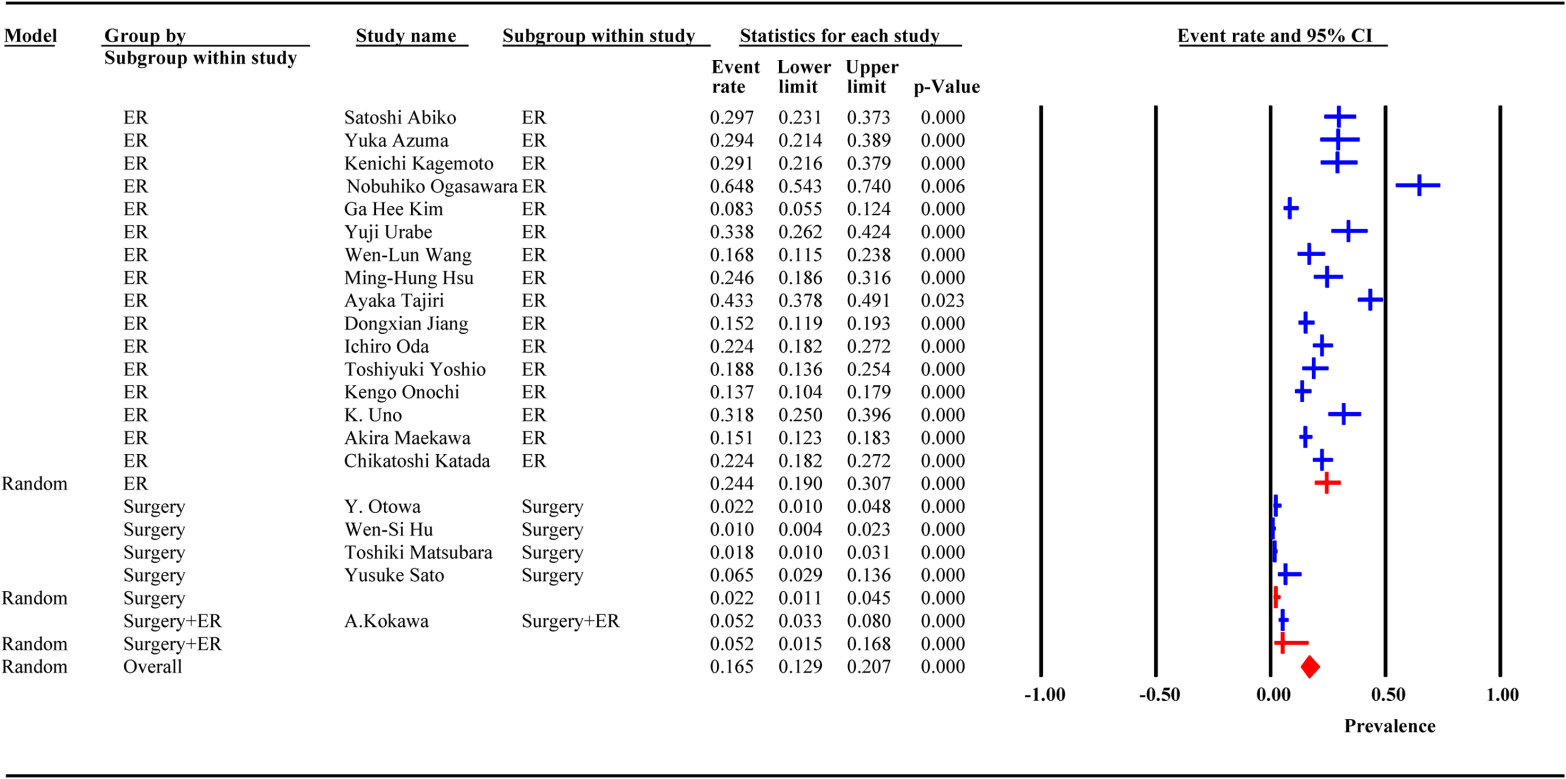
Forest plot of incidence of metachronous esophageal carcinoma (subgroup: removal method).

Incidence data were extracted from 21 studies. Heterogeneity was high among the 3 subgroups. The random effect model analysis showed that the pooled cumulative incidence of MESCC was 25.7% (95% CI: 19.2%, 32.2%) after ER, 1.6% (95%CI: 0.8%, 2.4%) after surgery, and 5.2% (95% CI: 3.1%, 7.9%) after surgery or ER. The overall pooled MESCC occurrence was 20.3% (95% CI: 13.8%, 26.8%), as shown in **Figure 4**. None of the predetermined subgroup analysis variables effectively controlled for heterogeneity shown in **Table 6**.

**Table 6:** Subgroup analysis of the incidence of metachronous esophageal carcinoma.

Subgroup analysis variables	Number of studies	Heterogeneity		Prevalence (%, 95%CI)
		I^2^	P-value	
Removal method				
ER	16	93.39%	0.000	24.3 (22.9, 25.8)
Surgery	4	72.71%	0.012	2.2 (1.5, 3.1)
Surgery/ER	1	–	1.000	5.2 (3.3, 8.0)
Type of cohort study				
Prospective	5	72.50%	0.002	22.3 (19.8, 25.1)
Retrospective	16	96.76%	0.000	21.1 (19.6, 22.6)
Follow-up schedule				
Annual	2	0	0.626	30.6 (25.4, 36.4)
Biannual	7	93.75%	0.000	23.3 (21.1, 25.7)
Every6-12 months	9	97.33%	0.000	18.9 (17.2, 20.6)
–	3	96.57%	0.000	19.8 (15.4, 25.1)
Follow-up time				
<60 months	11	93.84%	0.000	21.4 (19.7, 23.3)
>60 months	7	97.32%	0.000	25.2 (23.0, 27.5)
–	3	17.00%	0.300	6.60 (5.00, 8.70)

ER, endoscopic resection; -, missing data.

Among 21 studies, 18 reported the duration of follow-up, of which 13 were presented as median and 5 as mean. The meta-regression analysis showed that the incidence of MESCC increased with an extended follow-up time. For each additional year of follow-up, the incidence increased by 0.20%. Due to the different presentation formats (median and mean) of the follow-up time, subgroup analysis was performed. In the median subgroup, the incidence of MESCC increased by 0.36% while the mean decreased by 0.2% for each additional year of follow-up. The predicted 5-year and 10-year incidence rates of MESCC were 12.65% (95% CI: -7.56%, 30.10%) and 13.65% (95% CI: -5.16%, 32.35%), respectively.

### 3.5 Location of SPC after ER and surgery

**Figure 5:**
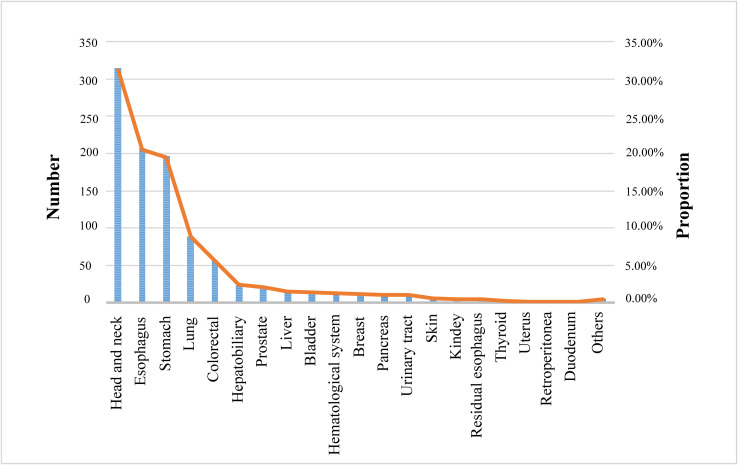
Distribution of second primary cancers.

Twelve studies described the distribution of SPC, of which SPC occurring after ER or surgery was diagnosed in more than 20 organs or systems of patients. The head and neck (31.12%) were the most common locations for the distribution of SPC, followed by the esophagus (20.52%), stomach (19.43%), lung (8.82%), and colorectal (5.65%), as shown in **Figure 5**.

### 3.6 Risk-of-bias assessment

**Figure 6:**
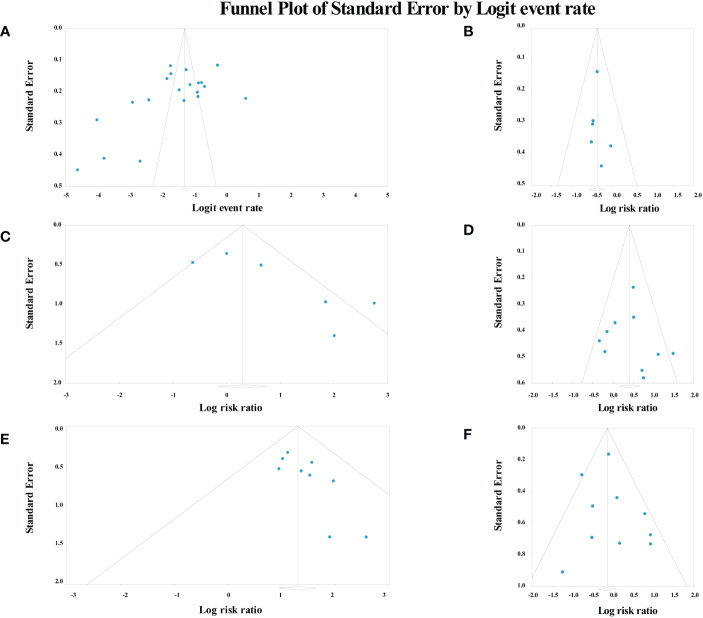
Funnel plot of risk factors and incidence analysis. **(A)** the incidence of metachronous esophageal carcinoma; **(B)** age; **(C)** alcohol consumption; **(D)** smoking; **(E)** Lugol-voiding lesions; **(F)** gender.

The Egger test analysis showed no publication bias for the following risk factors, including smoking (p=0.38), gender (p=0.28), age (p=0.41), history of multiple other cancer (p=0.35), smoking after ER (p=0.46), alcohol consumption after ER (p=0.37), tumor location (p=0.28), tumor depth (p=0.43) and macroscopic type (p=0.073). Conversely, alcohol consumption (p=0.025) and LVLs (p=0.014) showed publication bias. Also, the incidence of MESCC had publication bias (p=0.012). The funnel plots are summarized in **Figure 6**.

## 4 Discussion

This study involves 3 parts, risk factors for MESCC, incidence of MESCC and distribution of SPC. To our knowledge, this study is the first to examine the risk factors and incidence for MESCC after endoscopic or surgical resection of EC.

In terms of risk factors, 13 potential risk factors for MESCC were analyzed, among which 7 risk factors including smoking, gender, age, alcohol consumption, history of multiple other cancer, smoking after ER, and alcohol consumption after ER were categorized as demographic, individual factors. In contrast, LVLs, tumor location, macroscopic type, tumor depth, tumor size and lymphovascular invasion were categorized as endoscopic and histological factors. All factors except tumor size and lymphovascular invasion were quantitatively analyzed and the heterogeneity of smoking, gender, alcohol consumption, alcohol consumption after ER and macroscopic type were higher than 30%. After sensitivity analysis and subgroup analysis, factors of smoking, age, and history of multiple other cancer in demographic, individual category and the factor of LVLs in endoscopic and histological category were quantitatively determined to be associated with the development of MESCC. Smoking increases cancer risk by damaging DNA, producing carcinogens, and affecting immune function. The articles suggested that smoking may affect the prognosis and risk of SPC in patients with EC (32, 33), which is consistent with the conclusion of this review. Some studies suggested that the incidence of MESCC was not related to age (7, 8). However, some studies have also shown that age may have a protective factor effect on the development of MESCC (21, 34), which is consistent with the conclusion of the review. This is mainly because, with increasing age, patients pay more attention to their health, dietary habits, postoperative treatment, and follow-up. Therefore, regardless of age, patients should maintain a good lifestyle and adhere to regular follow-ups after EC removal to reduce the risk of developing MESCC. In this study, LVLs were also identified as a risk factor for developing MESCC after EC removal because LVLs are essential markers for predicting the early reoccurrence of EC in the residual mucosa after ER. Previous studies have shown that patients with LVLs (5, 10, 19, 35, 36) or other types of cancers in the past (21, 24) are prone to reoccurring EC and MESCC after removal of EC, respectively. In the qualitative analysis of factors, LVLs were identified as “definite factors,” history of multiple other cancer as a “likely factor,” while smoking, gender, age, alcohol consumption, and alcohol consumption after ER as “unclear factors” and the others as “not a risk factor.”. However, the negative results for other poorly documented risk factors do not invalidate them as risk factors related to the incidence of MESCC.

Based on the identified risk factors including smoking, age, history of multiple other cancer and LVLs, high-risk populations are found timely and strictly monitored and followed up to prevent patients from ending dangerous outcomes. Age, history of multiple other cancer, LVLs are unchangeable health risk factors, and smoking is changeable health risk factor. For unchangeable risk factors, routine investigation and surveillance are necessary. Once risk factors are found, attention should be paid to this population. People with young age, history of multiple other cancer, or detected LVLs were identified as high-risk populations. Timely investigating the age of patients, previous history of cancer and monitoring the number of LVLs are of great significance for individuals who will develop MESCC after removal of EC. Detected LVLs are explicitly correlated with the incidence of MESCC. Immediate monitoring of MESCC when LVLs occur facilitates timely physician treatment and patient recovery. For changeable risk factors, education and behavior correction are important. Smoking less or even no smoking should be actively advocated. Although this study showed that the postoperative smoking of the patient had nothing to do with the incidence of MESCC, which may be related to the lack of included studies, there is absolutely no harm in stopping smoking.

In terms of the incidence of MESCC, the overall pooled MESCC occurrence was 20.3%. The MESCC occurrence after ER, surgery and surgery or ER was 25.7%, 1.6% and 5.2%, respectively. None of the previously identified variables for subgroup analysis reasonably explained the sources of heterogeneity. Surgery has a lower incidence of MESCC suggested that the method of removal could affect the incidence of MESCC, which provided a reference for doctors to choose the removal method. This is consistent to some extent with the findings of Bestetti et al. on early gastric cancer, where ER was associated with higher risk of recurrence compared with surgery (37). The lower incidence of MESCC may be related to more complete surgical resection of the lesion. Compared with surgery, ER has more stringent conditions, such as the absence of lymph node metastasis, shallow depth of tumor involvement, etc. Currently, preoperative clinical staging relies on imaging which is not as sensitive as postoperative pathological biopsy, which may lead to inaccuracy on some patients and requirement for a sophisticated and mature preoperative assessment method. However, the small number of included papers with surgery as the treatment did have an impact on the reliability of the results. The meta-regression prediction of the incidence of MESCC at 5 and 10 years was 12.65% and 13.65%, respectively. This result shows that the incidence of MESCC was high in the first 5 years after operation reaching half of the overall pooled incidence and decreasing afterward. Thus, focusing on the first 5 years after operation is critical, which will provide the basis for clinical prevention and practice.

In terms of distribution of SPC, this review identified the most common location of SPC of the EC after ER or surgery was head and neck, followed by the esophagus. Therefore, in clinical practice, physicians should be concerned about the occurrence of MESCC and the emergence of head and neck carcinoma, EC, gastric carcinoma, and lung carcinoma in EC patients after operation, which requires physicians to enhance the detection of tumor markers at these sites.

Governments should not only invest more in primary health services to enable faster diagnosis and timely treatment, such as screening and follow-up of high-risk populations, but also invest more in scientific research to enable science to guide clinical practice more accurately. Clarifying the responsibilities of primary medical staff, actively carrying out community population education, and consolidating the important position of primary medical institutions in the entire medical system are of great significance for education, supervision, monitoring. Once suspected signs of MESCC or SPC are found, they will be referred to a higher-level hospital immediately.

Policy makers need to develop a comprehensive evaluation system for doctors which abandons the principle of “leaving the hospital is over” and values the lifelong management of the patients. The monitoring and long-term follow-up of patients should be included in the evaluation system of doctors, including the monitoring of risk factors and SPC, especially in the first 5 years after operation. Hospital staff should be assigned to monitor clinicians’ prescriptions, such as whether clinicians regularly detect LVLs of patients. The cost of diagnosis and treatment of EC should be covered by medical insurance to increase the compliance of patients.

Researchers should focus on both clinical research and basic theoretical research. Designing large, rigorously designed cohort studies to verify and validate the risk factors of MESCC and the increased incidence of MESCC per additional year. Research on the relationship between postoperative factors of EC and MESCC is encouraged and expected. It is the most direct and effective to determine the basic molecular biology causes of MESCC after operation of EC, but it is also the most arduous and painstaking.

Some limitations exist in the review. Firstly, the number of studies and sample size included limited the reliability and outreach of the results. In particular, there was a lack of monitoring of postoperative behavior of EC patients. Secondly, although subgroup analysis was performed in predicting annual increases in incidence, differences in the follow-up schedule and time led to bias in the results. Thirdly, even if subgroup analysis was performed in risk factor analysis, different data types would inevitably affect the results. Fourthly, most of the data came from male. It is well known that male and female have distinct differences in physical states, lifestyle habits, susceptibility genes and other aspects, even though the results of this study indicated that gender had nothing to do with the occurrence of MESCC. What is more, all of studies were from Asia, which lack of data support from Western research. Last but not least, due to the nature of the review articles, it is not possible to determine whether the risk factors identified in this study are independent risk factors.

## Data availability statement

The original contributions presented in the study are included in the article/**Supplementary Materials**. Further inquiries can be directed to the corresponding author.

## Author contributions

JD, ZB, and XW contributed to the conception and design of the study. JD and ZB organized the database. JD and TL performed the statistical analysis. JD and ZB wrote the first draft of the manuscript. JD, ZB, HZ, JZ, and RX wrote sections of the manuscript. All authors read, reviewed, and approved the final version.
