# Supplementary material for: Risk factors for metachronous esophageal squamous cell carcinoma after endoscopic or surgical resection of esophageal carcinoma: a systematic review and meta-analysis
Source: Front Oncol. 2023 Sep 14;13:1241572. doi: 10.3389/fonc.2023.1241572 (PMC10540085; doi:10.3389/fonc.2023.1241572)
Supplement: Supplementary file 1 [file DataSheet_1.docx]

**Search Strategies**

**PubMed**

1. "epidemiologic factors" [Mesh]
2. "risk factors" [Mesh]
3. "risk factor" [Title/Abstract]
4. "risk factors" [Title/Abstract]
5. "influence factor" [Title/Abstract]
6. "influence factors" [Title/Abstract]
7. "predictive factor" [Title/Abstract]
8. "predictive factors" [Title/Abstract]
9. "epidemiologic factor" [Title/Abstract]
10. "epidemiologic factors" [Title/Abstract]
11. OR/ #1 - #10
12. "endoscopic submucosal dissection" [Title/Abstract]
13. "ESD" [Title/Abstract]
14. "EMR" [Title/Abstract]
15. "endoscopic mucosal resection" [Title/Abstract]
16. "endoscopic resection" [Title/Abstract]
17. "esophagectomy" [Title/Abstract]
18. "oesophagectomy" [Title/Abstract]
19. "surgery" [Title/Abstract]
20. "endoscopy" [Mesh]
21. "endoscopic Mucosal Resection" [Mesh]
22. "esophagectomy" [Mesh]
23. OR/ #12 - #22
24. "metachronous" [Title/Abstract]
25. "second primary" [Title/Abstract]
26. "secondary" [Title/Abstract]
27. "Neoplasms, Second Primary"[Mesh]
28. OR/ #24 - #27
29. "esophageal" [Title/Abstract]
30. "esophagus" [Title/Abstract]
31. "Esophageal Neoplasms" [Mesh]
32. OR/ #29 - #31
33. #11 AND #23 AND #28 AND #32

**Embase**

1. 'risk factor': ti,ab,kw
2. 'risk factors': ti,ab,kw
3. 'influence factor': ti,ab,kw
4. 'influence factors': ti,ab,kw
5. 'predictive factor': ti,ab,kw
6. 'predictive factors': ti,ab,kw
7. 'epidemiologic factor': ti,ab,kw
8. 'epidemiologic factors': ti,ab,kw
9. 'risk factor'/exp
10. 'risk factor'
11. OR/ #1 - #10
12. 'endoscopic submucosal dissection': ti,ab,kw
13. 'ESD': ti,ab,kw
14. 'EMR': ti,ab,kw
15. 'endoscopic resection': ti,ab,kw
16. 'endoscopic mucosal resection': ti,ab,kw
17. 'oesophagectomy': ti,ab,kw
18. 'esophagectomy': ti,ab,kw
19. 'endoscopic mucosal resection'/exp
20. 'endoscopy'/exp
21. 'surgery'/exp
22. 'esophagectomy'/exp
23. OR/ #12-#22
24. 'metachronous': ti,ab,kw
25. 'second primary': ti,ab,kw
26. 'secondary': ti,ab,kw
27. 'second primary neoplasm'/exp
28. OR/ #24 - #27
29. 'esophageal': ti,ab,kw
30. 'esophagus': ti,ab,kw
31. 'esophagus tumor'/exp
32. OR/ #29 - #31
33. #11 AND #23 AND #28 AND #32

**Cochrane library**

1. 'risk factor': ti,ab,kw
2. 'risk factors': ti,ab,kw
3. 'influence factor': ti,ab,kw
4. 'influence factors': ti,ab,kw
5. 'predictive factor': ti,ab,kw
6. 'predictive factors': ti,ab,kw
7. 'epidemiologic factor': ti,ab,kw
8. 'epidemiologic factors': ti,ab,kw
9. MeSH descriptor: [risk factors] explode all trees
10. MeSH descriptor: [epidemiologic factors] explode all trees
11. OR/ #1 - #10
12. 'endoscopic submucosal dissection': ti,ab,kw
13. 'ESD': ti,ab,kw
14. 'EMR': ti,ab,kw
15. 'endoscopic resection': ti,ab,kw
16. 'endoscopic mucosal resection': ti,ab,kw
17. 'oesophagectomy': ti,ab,kw
18. 'esophagectomy': ti,ab,kw
19. MeSH descriptor: [endoscopic mucosal resection] explode all trees
20. MeSH descriptor: [esophagectomy] explode all trees
21. MeSH descriptor: [Surgical Oncology] explode all trees
22. OR/ #12-#21
23. 'metachronous': ti,ab,kw
24. 'second primary': ti,ab,kw
25. 'secondary': ti,ab,kw
26. MeSH descriptor: [Neoplasms, Second Primary] explode all trees
27. OR/ #24 - #26
28. 'esophageal': ti,ab,kw
29. 'esophagus': ti,ab,kw
30. MeSH descriptor: [Esophageal Neoplasms] explode all trees
31. OR/ #29 - #30
32. #11 AND #22 AND #27 AND #31

**Web of Science**

1. TS="risk factor"
2. TS="risk factors"
3. TS="risk"
4. TS="influence factor"
5. TS="influence factors"
6. TS="predictive factor"
7. TS="predictive factors"
8. TS="epidemiologic factor"
9. TS="epidemiologic factors"
10. OR/ #1 - #9
11. TS="endoscopic submucosal dissection"
12. TS="endoscopic resection"
13. TS="endoscopic dissection"
14. TS="ESD"
15. TS="EMR"
16. TS=" oesophagectomy"
17. TS="endoscopic mucosal resection"
18. TS="esophagectomy"
19. TS="surgery"
20. OR/ #11 - #19
21. TS="metachronous"
22. TS="second primary"
23. TS="secondary"
24. OR/ #21 - #23
25. TS="esophageal"
26. TS="esophagus"
27. OR/ #25 - #26
28. #10 AND #20 AND #24 AND #27

**SCOPUS**

1. "risk factor": ti,ab,kw
2. "risk factors": ti,ab,kw
3. "risk": ti,ab,kw
4. "influence factor": ti,ab,kw
5. "influence factors": ti,ab,kw
6. "predictive factor": ti,ab,kw
7. "predictive factors": ti,ab,kw
8. "epidemiologic factor": ti,ab,kw
9. "epidemiologic factors": ti,ab,kw
10. OR/ #1 - #9
11. "endoscopic submucosal dissection": ti,ab,kw
12. "endoscopic resection": ti,ab,kw
13. "endoscopic dissection": ti,ab,kw
14. "ESD": ti,ab,kw
15. "EMR": ti,ab,kw
16. "oesophagectomy": ti,ab,kw
17. "endoscopic mucosal resection": ti,ab,kw
18. "esophagectomy": ti,ab,kw
19. "surgery": ti,ab,kw
20. OR/ #11 - #19
21. "metachronous": ti,ab,kw
22. "second primary": ti,ab,kw
23. "secondary": ti,ab,kw
24. OR/ #21 - #23
25. "esophageal": ti,ab,kw
26. "esophagus": ti,ab,kw
27. OR/ #25 - #26
28. #10 AND #20 AND #24 AND #27
